# Histone demethylase KDM7A reciprocally regulates adipogenic and osteogenic differentiation via regulation of C/EBPα and canonical Wnt signalling

**DOI:** 10.1111/jcmm.14126

**Published:** 2019-01-07

**Authors:** Xiaoyue Yang, Guannan Wang, Yi Wang, Jie Zhou, Hairui Yuan, Xiaoxia Li, Ying Liu, Baoli Wang

**Affiliations:** ^1^ NHC Key Lab of Hormones and Development, Tianjin Key Lab of Metabolic Diseases Metabolic Diseases Hospital & Institute of Endocrinology, Tianjin Medical University Tianjin China; ^2^ Stomatological Hospital, Tianjin Medical University Tianjin China; ^3^ College of Basic Medical Sciences Tianjin Medical University Tianjin China

**Keywords:** adipogenesis, CCAAT/enhancer binding protein, histone demethylase, osteogenesis, Wnt

## Abstract

Recent emerging evidences revealed that epigenetic methylation of histone and DNA regulates the lineage commitment of mesenchymal progenitor cells. This study was undertaken to delineate the actions of histone lysine demethylase 7A (KDM7A) on osteogenic and adipogenic differentiation. Kdm7a expression was up‐regulated in primary marrow stromal cells and established stromal ST2 line after adipogenic and osteogenic treatment. Silencing of endogenous Kdm7a in the cells blocked adipogenic differentiation whereas promoted osteogenic differentiation. Conversely, overexpression of wild‐type Kdm7a in the progenitor cells enhanced adipogenic differentiation whereas inhibited osteogenic differentiation. However, the effect of KDM7A on cell differentiation was largely attenuated when the point mutation was made that abolishes enzymatic activity of KDM7A. Mechanism investigations revealed that silencing of Kdm7a down‐regulated the expression of the CCAAT/enhancer binding protein α (C/EBPα) and secreted frizzled‐related protein 1 (Sfrp1). Chromatin immunoprecipitation (ChIP) assay revealed that KDM7A directly binds to the promoters of C/EBPα and Sfrp1 and removes the histone methylation marks H3K9me2 and H3K27me2. Furthermore, silencing of Kdm7a activated canonical Wnt signalling. Thereafter, activation of canonical Wnt signalling through silencing of Sfrp1 in ST2 attenuated the stimulation of adipogenic differentiation and inhibition of osteogenic differentiation by KDM7A. Our study suggests that KDM7A balances adipogenic and osteogenic differentiation from progenitor cells through epigenetic control of C/EBPα and canonical Wnt signalling and implicates that control of KDM7A action has an epigenetic perspective of curtailing metabolic disorders like osteoporosis.

## 1 INTRODUCTION

Marrow stromal cells (MSCs), also known as bone marrow‐derived mesenchymal progenitor cells, have the potential to differentiate into various kinds of cell types, eg, osteoblasts, chondrocytes, adipocytes and myoblasts.^1^ Normally, there exists a reciprocal balance between adipogenesis and osteogenesis,^2^, ^3^ which is sometimes impaired in various human disorders including ageing, osteoporosis and obesity, etc.^4^, ^5^ It is crucial to elucidate the mechanisms that fine tune the balance between osteogenic and adipogenic differentiation.

A number of critical signalling pathways are involved in regulating the osteogenic and adipogenic commitment of MSCs, including transforming growth factor‐β (TGF‐β)/bone morphogenetic protein (BMP) signalling, canonical Wnt signalling, Notch and Hedgehogs, etc.^6^, ^7^, ^8^, ^9^ Besides, multiple transcription factors are critical for the differentiation of MSCs into adipocytes or osteoblasts. While runt‐related transcription factor 2 (Runx2) and Osterix are required for osteogenic differentiation,^10^, ^11^ CCAAT element binding protein (C/EBP) members and peroxisome proliferator‐activated receptor γ (PPARγ) play a role in adipogenic differentiation of MSCs.^12^, ^13^

Epigenetic regulation of gene expression through histone modifications is attracting more attention as the player in lineage‐specific commitment of progenitor cells.^14^, ^15^, ^16^ Histone modifications occur usually at lysine and arginine residues, and may alter histone‐DNA binding affinities and the interactions of specific transcription factors with the promoters.^17^ Histone demethylases are the classes of enzymes that remove methyl groups in modified histone proteins. Although largely unknown, recent emerging evidences have shown that histone demethylases may exert essential regulatory functions in cell fate decision of MSCs.^18^ Ye et al have demonstrated that the histone demethylases KDM4B and KDM6B play a positive role in osteogenic commitment of MSCs at the expense of adipogenic differentiation.^19^ The mechanism investigations showed that KDM6B increased the levels of homeobox (HOX) genes by removing H3K27me3, whereas KDM4B promoted the expression of distal‐less homeobox (DLX) genes by removing H3K9me3.^19^ Of interest, H3K27me3‐ and H3K9me3‐positive MSCs of bone marrow were significantly increased in ovariectomized and ageing mice in parallel with the highly active adipogenesis.^19^ Furthermore, KDM5A and KDM2A have also been recognized as osteogenic regulators, both of which negatively regulated osteogenic differentiation of MSCs.^20^, ^21^ The mechanism exploration revealed that KDM5A decreased the expression level of Runx2 through removing H3K4me3 levels from the promoter of Runx2,^20^ whereas KDM2A enhanced secreted frizzled‐related protein 2 (Sfrp2) transcription by decreasing histone H3K4 and H3K36 methylation at the Sfrp2 promoter.^21^

The histone lysine(K)‐specific demethylase 7 (KDM7) subfamily is an emerging class of transcriptional coactivators that consists of three members, KDM7A, KDM7B and KDM7C. While KDM7B plays an essential role in neuronal differentiation, craniofacial development and tumour growth,^22^, ^23^ KDM7C is involved in various biological processes including osteogenesis and adipogenesis. In osteoblasts, KDM7C promotes DNA binding of Runx2 by directly demethylating mouse Runx2 at Lys245 or human Runx2 at Lys238, rather than by demethylating histones on Runx2 target genes.^24^ During adipogenesis, KDM7C physically interacts with C/EBPα and C/EBPδ and epigenetically boosts the C/EBP‐driven expression of adipogenic factors.^25^

Histone lysine demethylase 7A (KDM7A), also known as JHDM1D, is a member of the plant homeodomain (PHD) finger protein (PHF) family of PHD‐ and JmjC domain‐containing histone demethylases. It is able to catalyse the removal of di‐methylation marks H3K9m2 and H3K27m2 on the promoters of target genes,^26^ and through this, regulates fibroblast growth factor‐4 (FGF‐4) expression and neural differentiation.^27^ KDM7A also functions as a potential tumour suppressor through blocking tumour growth and angiogenesis.^28^ Up to now, it remains unknown if KDM7A regulates adipogenic and osteogenic commitment of mesenchymal stem cells.

In this study, we identified KDM7A as a player in adipogenic and osteogenic differentiation from progenitor cells. Mechanism studies revealed that this is based upon the stimulation of C/EBPα and Sfrp1 transcription as a result of the removal of the repressive H3K9me2 and H3K27me2 marks by KDM7A from the promoter regions of C/EBPα and Sfrp1.

## 2 MATERIALS AND METHODS

### 2.1 Cell cultures

Stromal ST2 cells were obtained from Riken Cell Bank (Tsukuba, Japan), and maintained in DMEM containing 10% FBS. Bone marrow stromal cells (BMSCs) were isolated from femurs and tibias of 4‐week‐old C57BL/6J mice and cultured in αMEM containing 10% foetal bovine serum. For adipogenic differentiation, confluent cells were cultured in adipogenic medium (a‐MEM containing 10% FBS, 0.5 μmol/L dexamethasone, 0.25 mmol/L methylisobutylxanthine, 5 μg/mL insulin and 0.5 mmol/L indomethacin) for 72 hours, followed by treatment for an additional 48 hours with 5 μg/mL insulin alone. For osteogenic differentiation, 80% confluent cells were cultured in osteogenic medium (αMEM containing 10% FBS, 50 μg/mL ascorbic acid and 5 mmol/L β‐glycerophosphate) for 3 days followed by RNA and protein isolation, or for 14 days followed by alkaline phosphatase (ALP) staining.

### 2.2 Quantitative RT‐PCR

RNA was extracted using a total RNA isolation kit (Gmbiolab, Taiwan). After reverse transcription with 1 μg of the total RNA and random primers, the cDNA was PCR‐amplified on a real‐time PCR cycler using a SYBR Green fluorescence PCR kit (Sangon Biotech, Shanghai, China) with gene specific primers. RT‐PCR amplifications were carried out for one cycle of 95°C for 10 minutes, followed by 40 cycles of 95°C for 10 seconds, 57°C for 10 seconds and 72°C for 10 seconds. β‐actin was used as internal control. The expression levels of target genes were measured by the comparative Ct (ΔΔCt) method. The sequences of the primers are listed in Table S1.

### 2.3 Constructs and transfections

The expression construct of Kdm7a was obtained from Origene (Rockville, MD, USA). A mutant mouse Kdm7a construct with H282A mutation that abolishes the enzymatic activity of KDM7A^26^ was made by using a mutagenesis kit (Vazyme Biotech, Nanjing, China). For the Kdm7a loss of function studies, we transfected ST2 cells with either 30 nmol/L Kdm7a siRNA or negative control siRNA (Genepharma, Shanghai, China) using lipofectamine RNAi‐Max (Gaithersburg, MD, USA). For the Kdm7a gain‐of‐function experiments, the ST2 cells were transfected with wild‐type or mutant Kdm7a expression plasmid, or the empty vector using Attractene transfection reagent (QIAGEN, Hilden, Germany) for 16 hours. Adipogenic or osteogenic induction was performed at appropriate confluence to allow the cells to differentiate.

For the co‐transfection studies, the Kdm7a expression plasmid and Sfrp1 siRNA were co‐transfected by using Attractene transfection reagent for 16 hours. At appropriate confluence of cells, adipogenic or osteogenic induction was performed to allow the cells to differentiate.

### 2.4 Lentiviral packaging and infection

To make the Kdm7a shRNA coding construct, two complementary strands targeting mouse Kdm7a gene were annealed and then cloned into the pLVX‐shRNA2 (Clontech, Palo Alto, CA, USA) at EcoRI/BamHI sites. The sequences of the strands are: sense, 5ʹ‐GATCCGTACTAAGTAA CTTTGAGGCTTCAAGAGAGCCTCAAAGTTACTTAGTATTTTTTACGCGTG‐3ʹ; antisense, 5ʹ ‐ AATTCACGCGTAAAAAATACTAAGTAACTTTGAGGCTCTCTTGAAGCCTCAAAGTT ACTTAGTACG‐3ʹ. The lentiviruses were packaged in 293T cells with the lentiviral packaging system (Jiman Biotech, Shanghai, China). Primary MSCs were infected with the viruses (multiplicity of infection = 20), followed by adipogenic or osteogenic treatment at appropriate cell confluence. The lentiviruses packaged with the empty vector were used as control.

### 2.5 Oil‐red O staining

Differentiated adipocytes were fixed in 4% paraformaldehyde, then washed with deionized water. After incubating with 60% isopropanol for 2 minutes, the cells were stained with oil‐red O solution (0.5% oil red O in isopropanol/water = 3:2) for 5 minutes. For oil‐red O quantification, isopropanol was added to dissolve the stain. Light absorbance was measured at 520 nm.

### 2.6 ALP staining

Differentiated osteoblasts were fixed in 4% paraformaldehyde for 10 minutes, and then stained with the 1‐Step nitroblue tetrazolium (NBT)/5‐bromo‐4‐chloro‐3‐indolyl phosphate (BCIP) staining kit (Sangon Biotech, Shanghai, China) for 15 minutes.

### 2.7 Western blot analysis

Proteins were separated by SDS‐PAGE and transferred onto nitrocellulose membranes. The membranes were incubated overnight with primary antibodies. The antibodies we used include rabbit antibodies by Abcam (Cambridge, MA, USA): anti‐osterix (polyclonal), anti‐C/EBPa (monoclonal), anti‐β‐catenin (monoclonal), anti‐opsteopontin (monoclonal) and anti‐ALP (monoclonal); rabbit antibodies by Cell Signalling Technology (Danvers, MA, USA): anti‐PPARγ (monoclonal), anti‐LRP6 (monoclonal) and anti‐Phospho‐LRP6 (polyclonal); mouse mAb by MBL (Nagoya, Japan): anti‐Runx2; rabbit polyclonal antibodies by Proteintech (Wuhan, China): anti‐aP2, and anti‐β‐actin; rabbit polyclonal antibody by SAB: anti‐H3K9me2 and rabbit polyclonal antibody by Bioworld: anti‐H3K27me2. The membranes were then incubated with the corresponding horseradish peroxide‐labeled IgG (1:3000) for 2 hours. Chemiluminescence reagent (Advansta, Menlo Park, CA, USA) was finally used to visualize the results.

### 2.8 Chromatin immunoprecipitation (ChIP) assay

Chromatin immunoprecipitation (ChIP) assay was performed using a kit from Cell Signaling Technology (Danvers, MA, USA) according to the supplier's instructions. After micrococcal nuclease digestion and sonication, cell lysates containing soluble chromatin were incubated overnight with 4 μg anti‐KDM7A, anti‐H3K9me2, anti‐H3K27me2 antibody or IgG. The de‐crosslinked DNA was used as templates to PCR amplify mouse C/EBPα‐ or Sfrp1‐specific sequences. The sequences of the primers are listed in supplemental Table S1.

### 2.9 Statistical analysis

Data are expressed as means ± SD. For the relative mRNA expression analysis, the means of the control groups were set to 1. Statistical analysis was performed with the independent *t* test or one‐way ANOVA. If the one‐way ANOVA result was significant, a post hoc comparison was performed with the least significant difference (LSD) test. A value of *P* < 0.05 indicated statistical significance.

## 3 RESULTS

### 3.1 Kdm7a was expressed in bone and adipose tissue and increased during osteoblast and adipocyte differentiation

**Figure 1:**
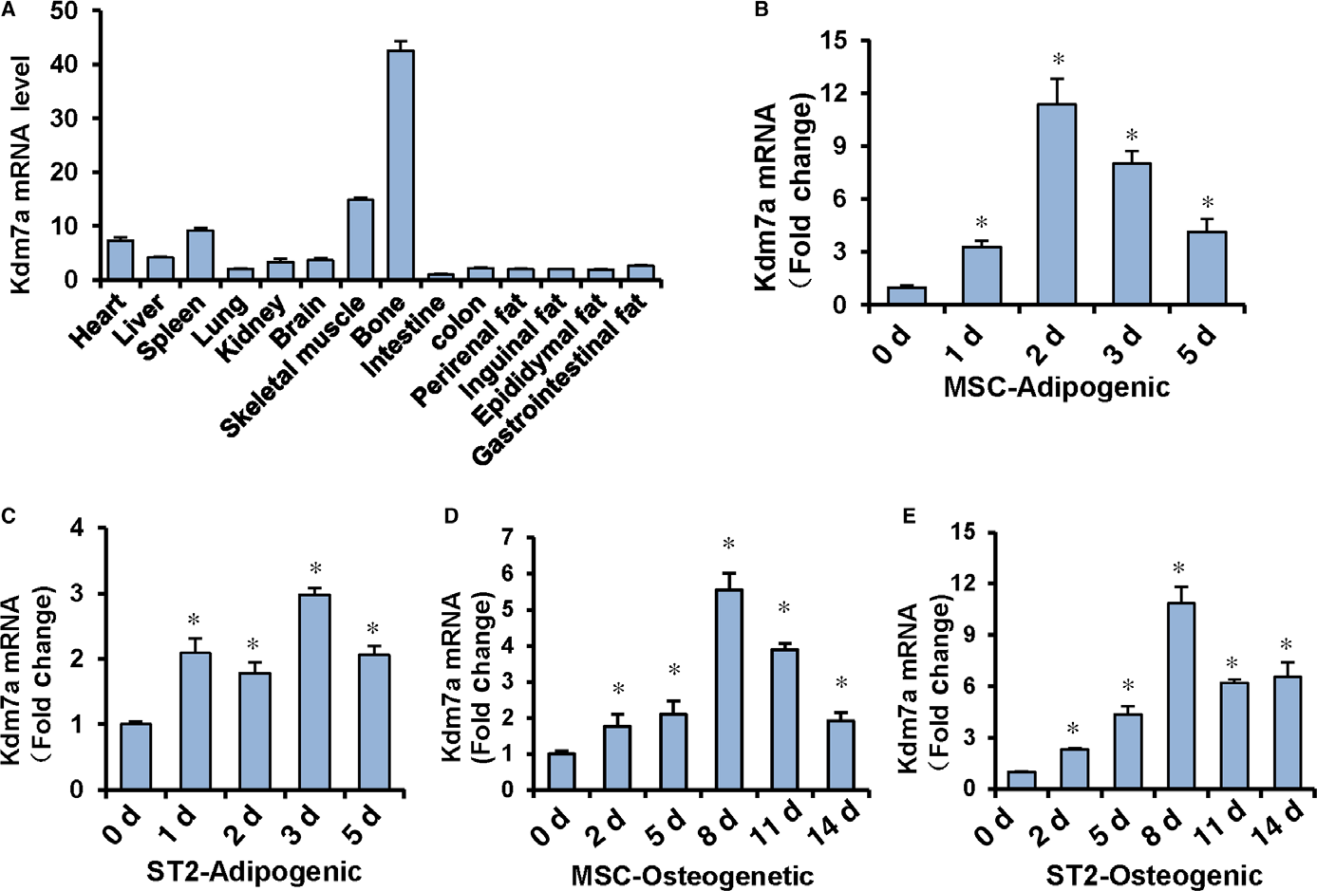
Histone lysine(K)‐specific demethylase 7A (Kdm7a) was increased during adipogenic and osteogenic differentiation. RT‐PCR showed that Kdm7a was expressed in various tissues of mice. The level of Kdm7a in intestine was set to 1 (A). qRT‐PCR revealed the up‐regulation of Kdm7a expression in primary MSCs (B) or ST2 (C) at indicated time points during adipogenesis. Kdm7a expression was up‐regulated in primary MSCs (D) or ST2 (E) during osteogenesis. The level of Kdm7a at d 0 was set to 1. Values are means ± SD (n = 3). *P < 0.05 vs vehicle treatment

We examined the expression levels of Kdm7a in various tissues in 1‐month‐old mice. Kdm7a mRNA was highly expressed in bone and skeletal muscle, and moderately expressed in spleen and heart. Kdm7a was expressed in relatively low level in other indicated tissues (Figure 1A). qRT‐PCR analysis revealed that Kdm7a expression was induced in primary MSCs and stromal ST2 cells after adipogenic treatment, peaking at d 2 in primary MSCs and d 3 in ST2, respectively (Figure 1B,C). After osteogenic treatment, the Kdm7a level was also increased, peaking at d 8 for primary MSCs and ST2 cells during osteogenic differentiation (Figure 1D,E). These results suggest that KDM7A has a regulatory role in adipogenic and/or osteogenic differentiation.

### 3.2 Silencing of endogenous Kdm7a in ST2 cells inhibited adipocyte formation and promoted osteoblast differentiation

**Figure 2:**
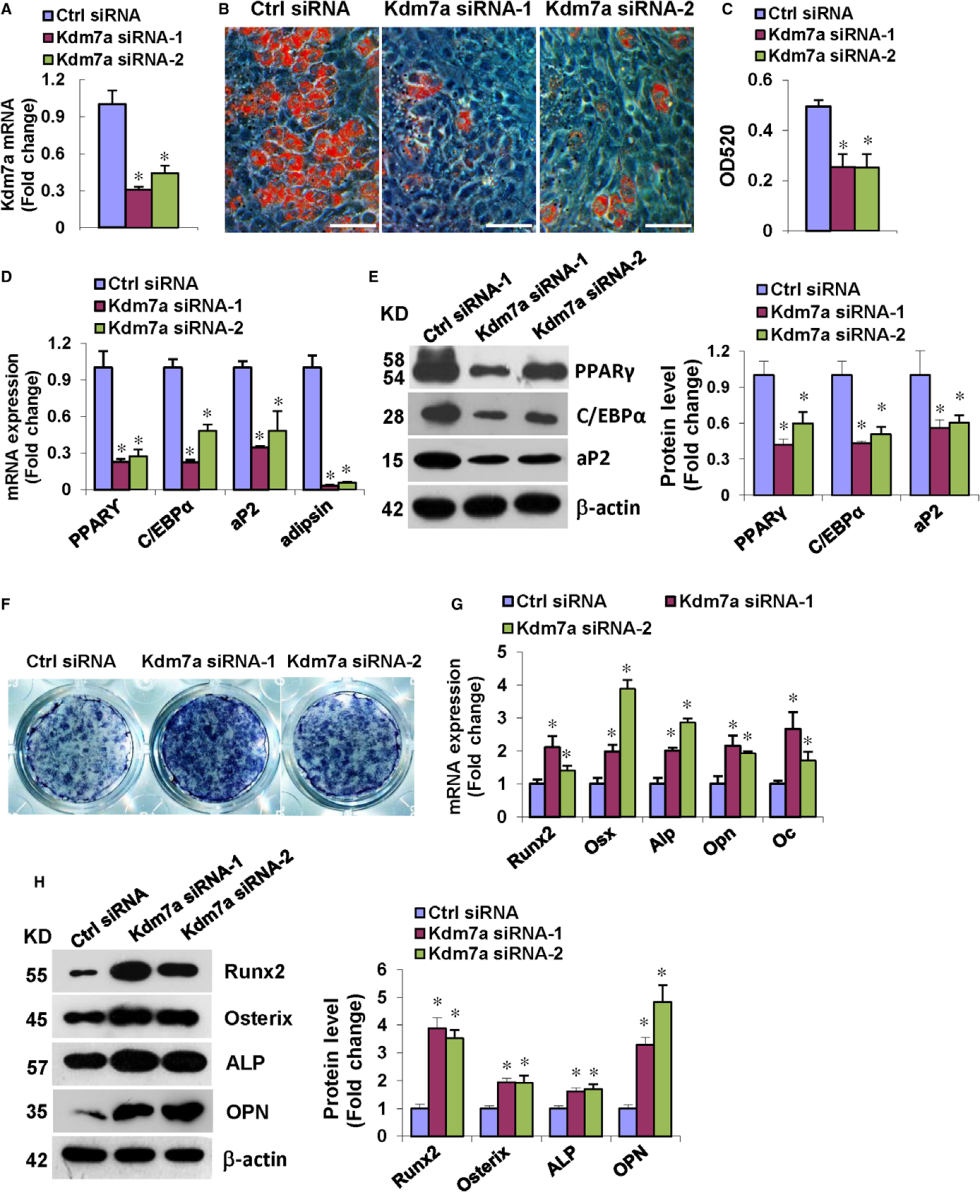
Silencing of Kdm7a in ST2 inhibited adipocyte formation and promoted osteoblast differentiation. qRT‐PCR verified the silencing of Kdm7a in ST2 (A). Kdm7a silencing reduced adipocyte formation after adipogenic treatment (B). Oil red O extracted with isopropanol was measured at OD520 (C). The mRNA levels of PPARγ, C/EBPα, aP2 and adipsin (D) and protein levels of PPARγ, C/EBPα and aP2 (E) were down‐regulated in Kdm7a silencing cells. Alkaline phosphatase staining was enhanced in Kdm7a silencing cells after osteogenic treatment (F). The mRNA levels of Runx2, Osterix, Alp, Osteopontin and Osteocalcin (G) and protein levels of Runx2, Osterix, ALP and Osteopontin (H) were increased in Kdm7a silencing cells. Image scale in (B): 200 μm. Values are means ± SD (n = 3). *P < 0.05 vs control siRNA

Two independent siRNAs targeting different coding regions of Kdm7a substantially down‐regulated the endogenous Kdm7a mRNA level in ST2 cells (Figure 2A). Functional experiments with these siRNAs inhibited adipogenic differentiation of ST2 cells. When compared to the cells transfected with control siRNA, the cells receiving Kdm7a siRNAs formed less adipocytes, as evidenced by OD520 measurement of dissolved oil red O stain (Figure 2B,C). The mRNA levels of PPARγ, C/EBPα, aP2 and adipsin were significantly reduced 48 hours after adipogenic treatment in Kdm7a‐silencing cells (Figure 2D). Consistently, Kdm7a‐silencing also led to the substantial decrease in the protein levels of PPARγ, C/EBPα and aP2 72 hours after adipogenic treatment (Figure 2E).

By contrast, the Kdm7a siRNAs positively affected the differentiation of ST2 cells into osteoblasts, revealed by enhanced ALP staining (Figure 2F). Consistently, the mRNA levels of Runx2, Osterix, Alp, Osteopontin (Opn) and Osteocalcin (Oc) were increased 72 hours after osteogenic treatment (Figure 2G). Moreover, the protein levels of Runx2, Osterix, ALP and Osteopontin were also substantially increased 72 hours after osteogenic treatment (Figure 2H).

### 3.3 Kdm7a overexpression in ST2 cells induced adipocyte formation and inhibited osteoblast differentiation and the effect is dependent on its demethylase activity

In order to further demonstrate whether KDM7A regulates adipogenic and osteogenic differentiation and whether this is dependent on its demethylase activity, we generated a wild‐type construct and a catalytically dead Kdm7a mutant in which histidine 282 was changed to alanine, disrupting the non‐haem metal binding site required for its demethylase activity.^26^, ^29^

**Figure 3:**
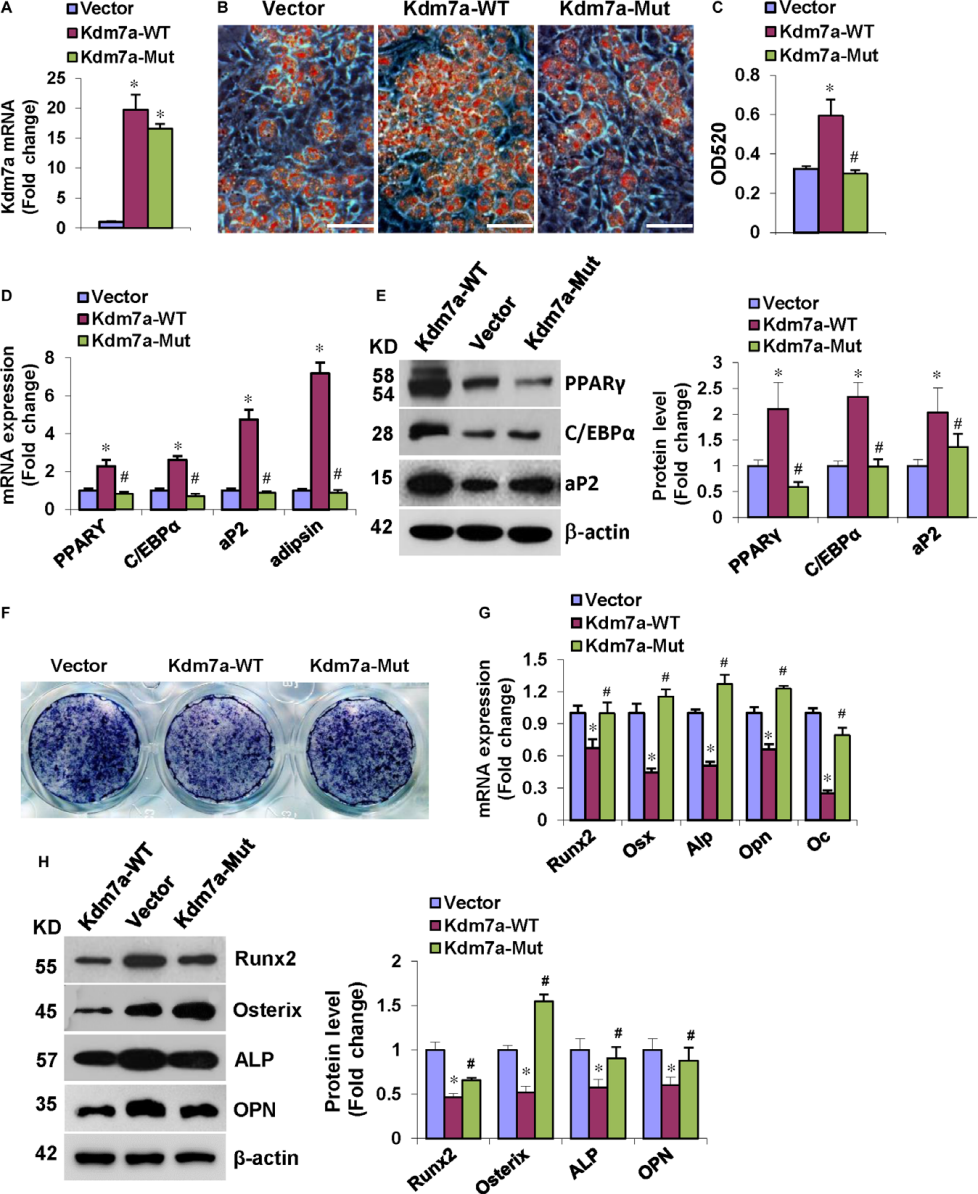
Kdm7a overexpression in ST2 induced adipocyte formation and inhibited osteoblast differentiation. qRT‐PCR verified the overexpression of Kdm7a in ST2 (A). Kdm7a‐WT transfection induced adipocyte formation after adipogenic treatment while the effect of Kdm7a‐Mut was attenuated (B). Oil red O extracted with isopropanol was measured at OD520 (C). Kdm7a‐WT transfection up‐regulated the mRNA (D) and protein (E) levels of adipogenic factors while the effect of Kdm7a‐Mut was attenuated. Kdm7a‐WT overexpression blunted ALP staining of differentiated osteoblasts while the inhibitory effect was attenuated in Kdm7a‐Mut expressing cells after osteogenic treatment (F). The mRNA (G) and protein (H) levels of osteogenic factors were decreased in Kdm7a‐WT expressing cells while the inhibitory effect was attenuated in Kdm7a‐Mut expressing cells. Image Scale in (B): 200 μm. Values are means ± SD (n = 3). *P < 0.05 vs Vector, ^#^ *P* < 0.05 vs Kdm7a‐WT

The overexpression of wild‐type or mutant Kdm7a in ST2 cells was verified with qRT‐PCR (Figure 3A). In presence of adipogenic medium, wild‐type Kdm7a overexpression significantly increased the number of differentiated adipocytes, while the effect was largely attenuated when we overexpressed mutant Kdm7a (Figure 3B,C). Accordingly, wild‐type Kdm7a induced the mRNA and protein levels of adipogenic transcription factors and marker genes 48 and 72 hours, respectively, after adipogenic treatment, whereas the effect of mutant Kdm7a was largely attenuated (Figure 3D,E).

Conversely, in the presence of osteogenic medium, wild‐type Kdm7a overexpression in ST2 cells inhibited osteoblast differentiation whereas the effect was largely attenuated when the mutant Kdm7a was overexpressed, as evidenced by ALP staining (Figure 3F). Consistently, wild‐type Kdm7a decreased the mRNA and protein levels of osteogenic factors 72 hours after osteogenic treatment, whereas the effect of mutant Kdm7a was largely attenuated (Figure 3G,H). These data suggest that the demethylase function of KDM7A is required for stimulation of adipogenic differentiation and suppression of osteogenic differentiation.

### 3.4 Kdm7a knock‐down in primary MSCs inhibited adipogenic differentiation and stimulated osteogenic differentiation

**Figure 4:**
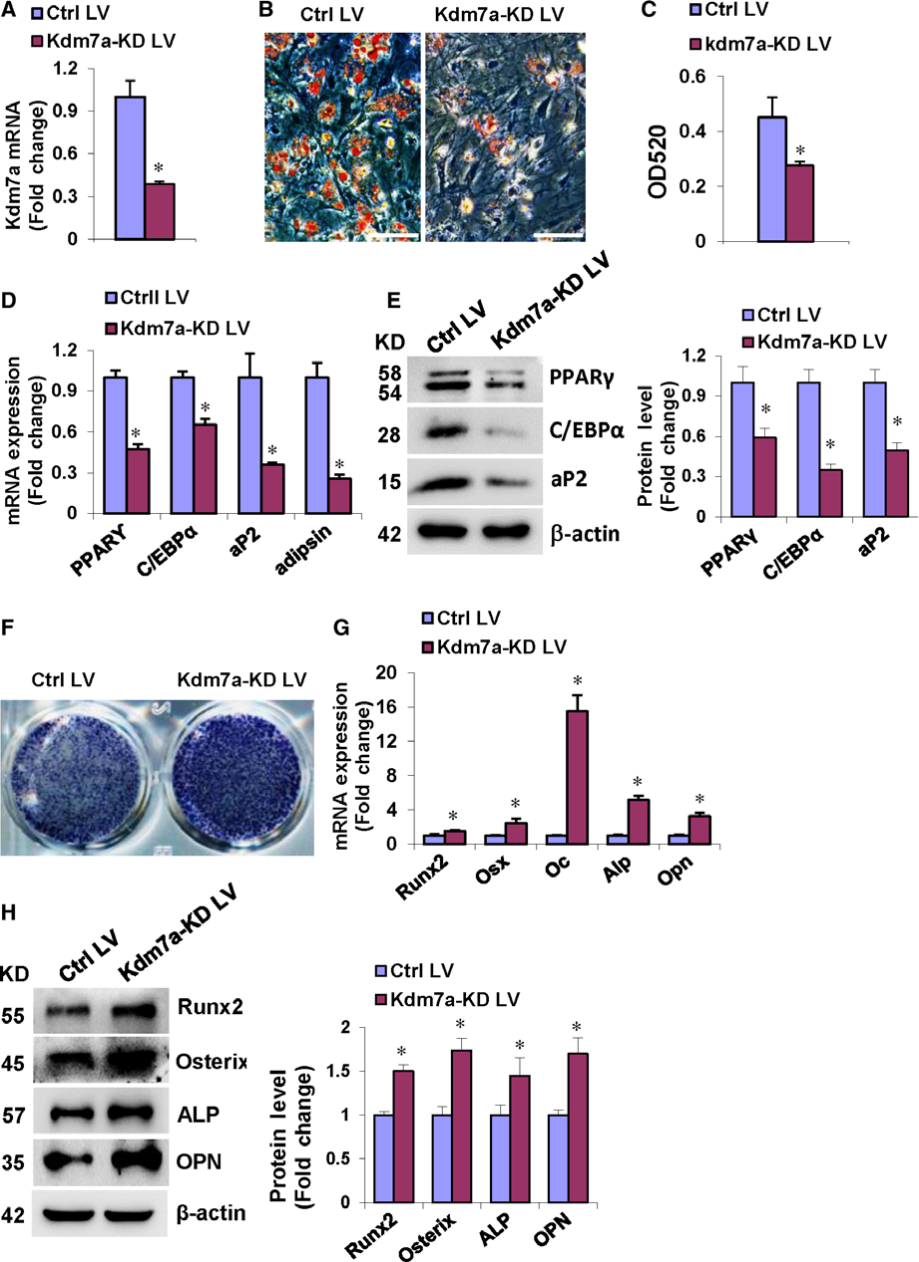
Kdm7a knock‐down in primary MSCs inhibited adipogenic differentiation and stimulated osteogenic differentiation. Primary mouse MSCs were infected with the Kdm7a shRNA virus. qRT‐PCR revealed Kdm7a silencing following infection (A). After adipogenic treatment, Kdm7a silencing blunted adipogenic differentiation (B, C), and decreased mRNA (D) and protein (E) levels of adipogenic factors. After osteogenic treatment, Kdm7a silencing potentiated ALP staining (F), and increased mRNA (G) and protein (H) levels of osteogenic factors. Image scale in (B): 200 μm. Values are means ± SD (n = 3). *Significant vs control lentivirus, *P* < 0.05

To further demonstrate the role of KDM7A in lineage commitment of progenitor cells, we made the Kdm7a shRNA lentiviruses that substantially down‐regulated the endogenous Kdm7a mRNA level in primary MSCs (Figure 4A). When compared to control cells, the cells transduced with Kdm7a silencing lentiviruses formed less adipocytes, as evidenced by oil red O staining and quantification of the dissolved stain (Figure 4B,C). Consistently, the cells transduced with Kdm7a silencing lentiviruses had lower mRNA and protein levels of the adipogenic factors at 48 and 72 hours, respectively, after adipogenic treatment (Figure 4D,E).

Conversely, transduction of Kdm7a silencing lentiviruses stimulated the differentiation of primary MSCs into osteoblasts, as evidenced by enhanced ALP staining (Figure 4F). Consistently, the mRNA and protein levels of the osteogenic factors were significantly induced 72 hours after osteogenic treatment (Figure 4G,H).

### 3.5 KDM7A targets C/EBPα and canonical Wnt signalling to regulate ST2 cells differentiation through removing H3K9me2 and H3K27me2

**Figure 5:**
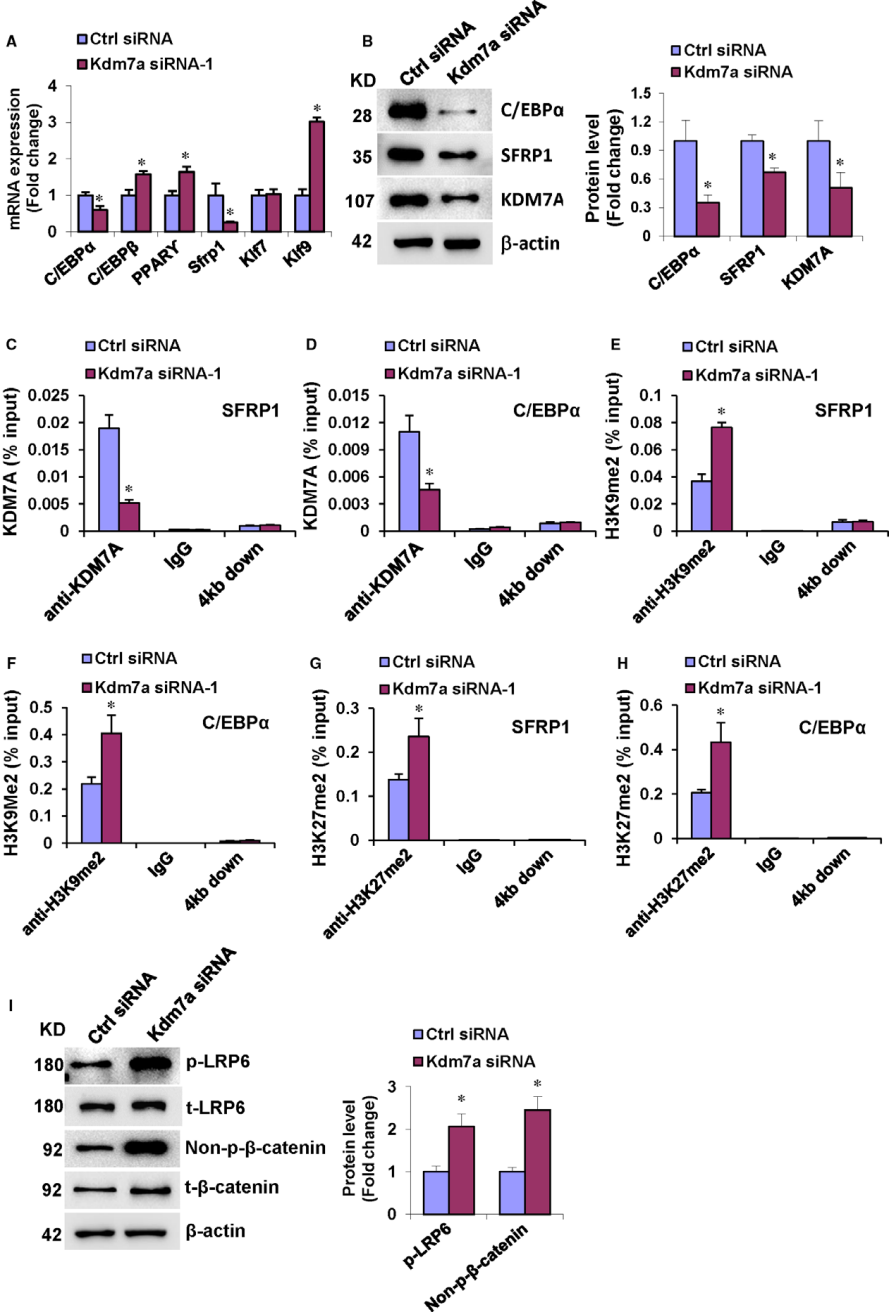
KDM7A targets C/EBPα and canonical Wnt signalling to regulate ST2 cells differentiation through removing H3K9me2 and H3K27me2. ST2 cells were cultured in DMEM containing 10% FBS without osteogenic or adipogenic treatment. Kdm7a siRNA transfection decreased the mRNA (A) and protein (B) levels of C/EBPα and Sfrp1. Kdm7a silencing reduced KDM7A occupancy on the promoters of C/EBPα and Sfrp1 (C, D). Kdm7a silencing increased binding of H3K9me2 and H3K27me2 to the promoter of C/EBPα and Sfrp1 (E‐H). Kdm7a silencing increased the protein levels of phopsho‐LRP6 and active form of β‐catenin (I). Values are means ± SD (n = 3). *P < 0.05 vs control siRNA

The commitment of MSCs is regulated by a complex and highly orchestrated gene expression program and many developmental signalling pathways. To further explore the mechanisms through which KDM7A regulates adipogenic and osteogenic differentiation, we tested whether the disruption of KDM7A affected the endogenous expression of key regulators of progenitor cells. Intriguingly, after depletion of Kdm7a, the basal levels of endogenous C/EBPα and Sfrp1 were significantly suppressed. By contrast, Kruppel‐like factor 7 (Klf7) was not affected, whereas C/EBPβ, PPARγ and Kruppel‐like factor 9 (Klf9) were even up‐regulated after silencing of Kdm7a (Figure 5A). Consistently, the protein levels of C/EBPα and SFRP1 were also decreased after silencing of Kdm7a (Figure 5B). These data imply that C/EBPα and SFRP1 may be regulated by KDM7A thereby playing roles in balancing the adipogenic and osteogenic differentiation of progenitor cells. To further examine how KDM7A controlled progenitor cell differentiation, and whether KDM7A regulated the differentiation through removing H3K9me2 or H3K27me2 marks, we carried out ChIP assay to assess the changes in histone methylation status at the promoter regions of those master regulator genes. The data showed that the knock‐down of Kdm7a reduced KDM7A binding to the promoters of C/EBPα and Sfrp1 in ST2 cells (Figure 5C,D). Moreover, the silencing of Kdm7a increased H3K9me2 and H3K27me2 levels at the promoters of both C/EBPα and Sfrp1 genes (Figure 5E‐H). As a control, we could not detect KDM7A occupancy on C/EBPα and Sfrp1 promoters 3‐4 kb downstream of the transcription start sites (Figure 5C,D), and the knock‐down of Kdm7a did not affect H3K9me2 and H3K27me2 at those regions (Figure 5E‐H). The data suggest that KDM7A may directly regulate C/EBPα and Sfrp1 expression. Furthermore, Western blotting showed that silencing of Kdm7a increased the protein levels of phopsho‐LRP6 and active form of β‐catenin (Figure 5I), suggesting that KDM7A is capable of inactivating canonical Wnt signalling.

### 3.6 Silencing of Sfrp1 in ST2 cells attenuated KDM7A stimulation of adipogenic differentiation and inhibition of osteogenic differentiation

**Figure 6:**
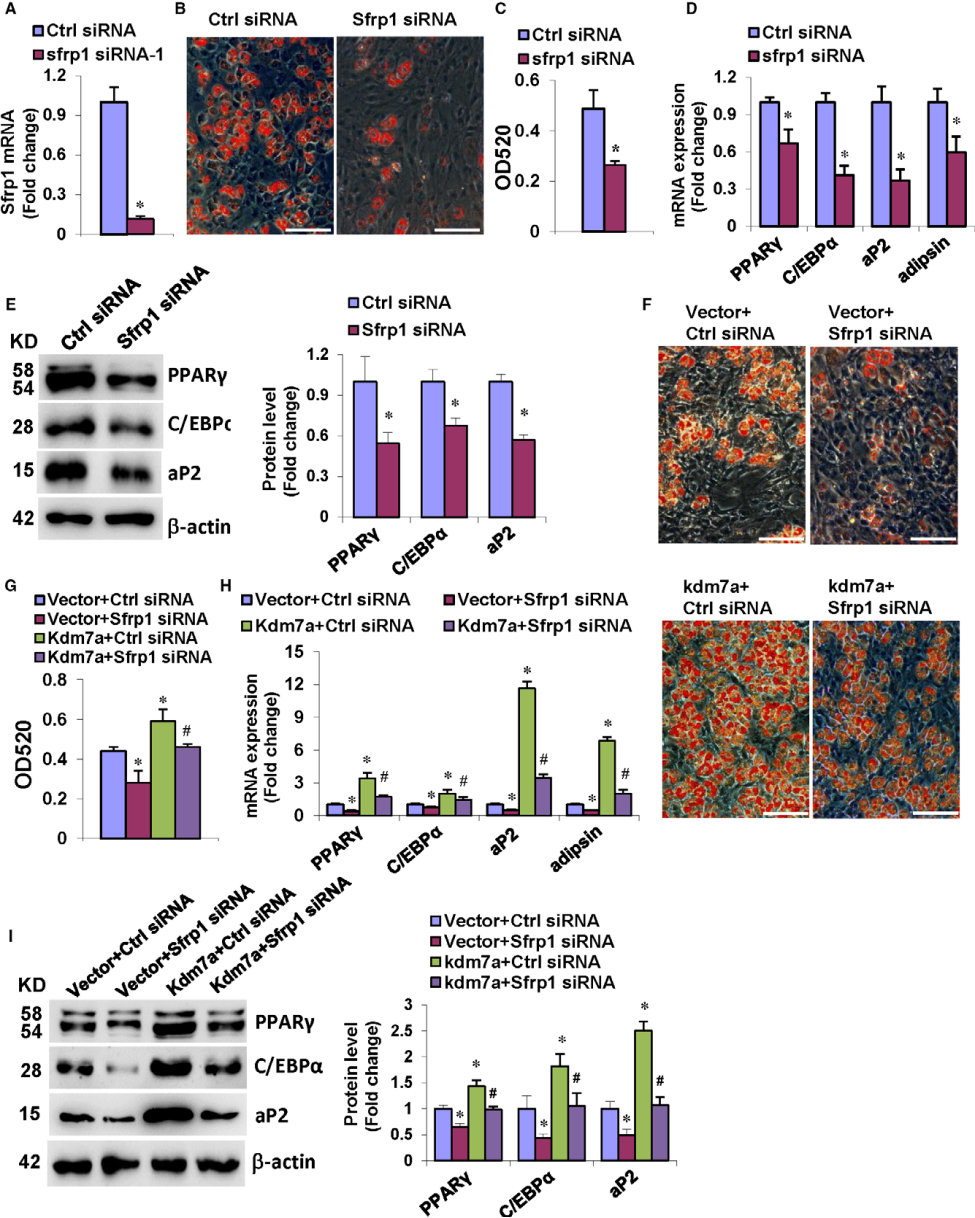
Silencing of Sfrp1 in ST2 cells attenuated KDM7A stimulation of adipogenic differentiation. qRT‐PCR verified the efficiency of Sfrp1 siRNA (A). After adipogenic treatment, Sfrp1 silencing blunted adipogenic differentiation (B, C), and decreased mRNA (D) and protein (E) levels of adipogenic factors. The co‐transfection of Sfrp1 siRNA along with Kdm7a expression construct attenuated adipogenic differentiation vs control siRNA plus Kdm7a (F, G). The mRNA (H) and protein (I) levels of adipogenic factors were decreased after co‐transfection of Sfrp1 siRNA and Kdm7a expression construct vs control siRNA plus Kdm7a. Image scale in (B, F): 200 μm. Values are means ± SD (n = 3). *Significant vs control siRNA plus vector, *P* < 0.05. ^#^Significant vs control siRNA plus Kdm7a

Secreted frizzled‐related protein 1 (SFRP1) is an extracellular attenuator of canonical Wnt signalling that plays important roles in both adipogenesis and osteogenesis. To further clarify whether SFRP1/Wnt signalling is involved in KDM7A regulation of adipogenesis and osteogenesis, we undertook KDM7A loss‐of‐function and gain‐of‐function studies. The efficacy of Sfrp1 siRNA to knock‐down Sfrp1 expression was demonstrated by using qRT‐PCR (Figure 6A). Silencing of Sfrp1 alleviated the adipogenic differentiation of ST2 cells, evidenced by oil red O staining of the adipocytes and quantification of dissolved stain (Figure 6B,C). Consistently, Sfrp1 silencing resulted in the reduction of the mRNA and protein levels of adipogenic factors 48 and 72 hours, respectively, after adipogenic treatment (Figure 6D,E).

Furthermore, the co‐transfection of the Kdm7a expression plasmid and Sfrp1 siRNA was performed. The data revealed that the ability of KDM7A to stimulate adipocyte formation was attenuated under the background of Sfrp1 silencing. The number of adipocytes formed in the cells co‐transfected with Kdm7a plasmid and Sfrp1 siRNA was less than in the cells receiving Kdm7a plasmid and control siRNA, although still more than in the cells receiving with Vector and Sfrp1 siRNA (Figure 6F,G). Consistently, the mRNA and protein levels of the adipogenic factors were decreased in the cells co‐transfected with Kdm7a plasmid and Sfrp1 siRNA vs the cells receiving Kdm7a plasmid and control siRNA (Figure 6H,I).

**Figure 7:**
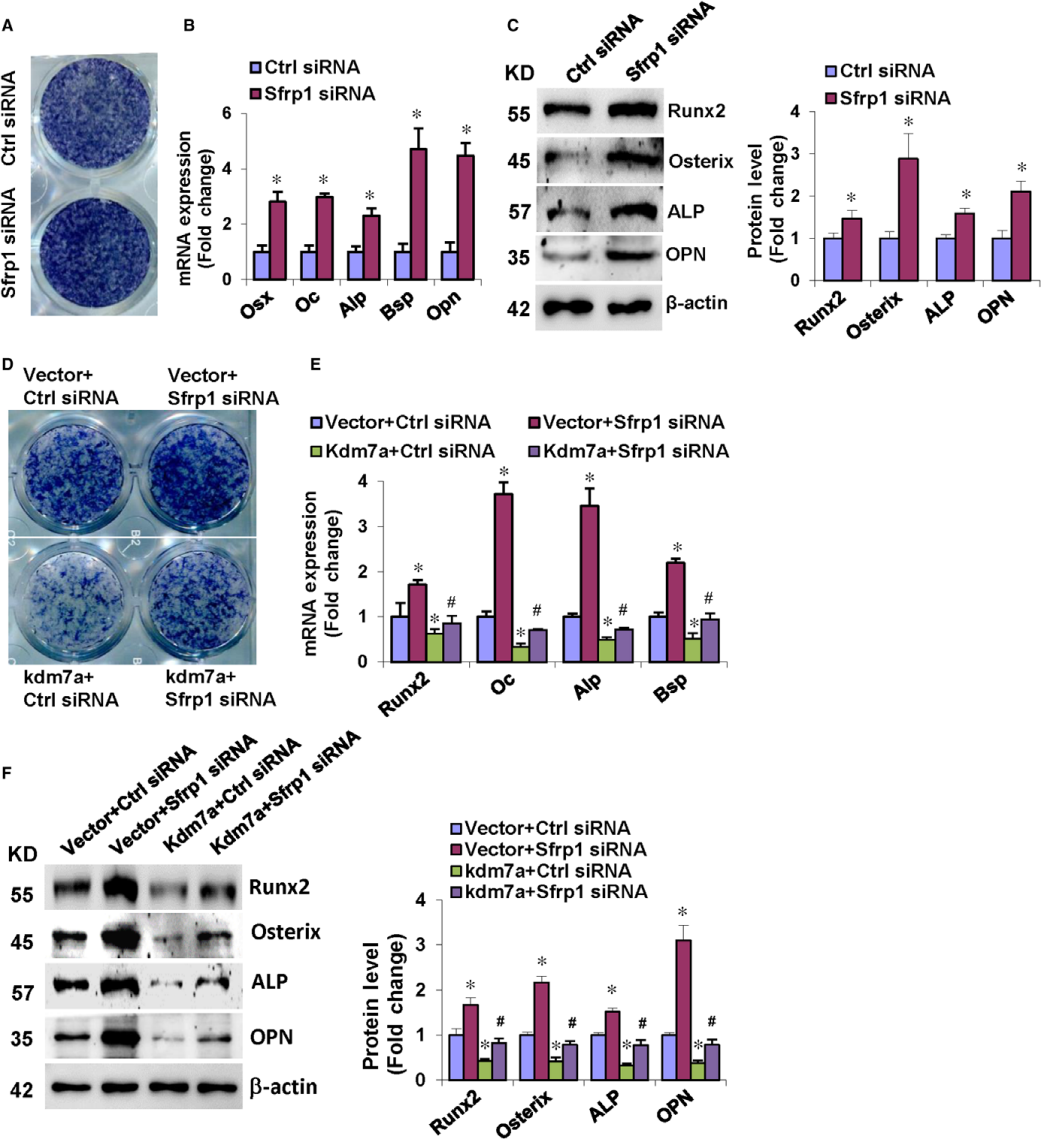
Silencing of Sfrp1 in ST2 cells attenuated KDM7A inhibition of osteogenic differentiation. Following osteogenic treatment, Sfrp1 silencing potentiated ALP staining (A), and increased mRNA (B) and protein (C) levels of osteogenic factors. The co‐transfection of Sfrp1 siRNA along with Kdm7a expression construct enhanced ALP staining in ST2 vs control siRNA plus Kdm7a (D). The mRNA (E) and protein (F) levels of osteogenic factors were increased after co‐transfection of Sfrp1 siRNA and Kdm7a expression construct vs control siRNA plus Kdm7a. Values are means ± SD (n = 3). *Significant vs control siRNA plus vector, *P* < 0.05. ^#^Significant vs control siRNA plus Kdm7a

By contrast, Sfrp1 silencing promoted the osteogenic differentiation of ST2 cells, evidenced by enhanced ALP staining (Figure 7A). Consistently, Sfrp1 silencing stimulated the mRNA and protein expression of the osteogenic factors 72 hours after osteogenic treatment (Figure 7B,C). We also performed Kdm7a gain‐of‐function studies under the background of Sfrp1 silencing. The data from the co‐transfection studies revealed that the potential of KDM7A to inhibit osteoblast differentiation was attenuated under the background of Sfrp1 silencing. The ALP staining of differentiated osteoblasts in the cells co‐transfected with Kdm7a plasmid and Sfrp1 siRNA was enhanced vs the cells receiving Kdm7a plasmid and control siRNA, although it was attenuated vs the cells receiving Vector and Sfrp1 siRNA (Figure 7D). Consistently, the mRNA and protein levels of the osteogenic factors were increased in the cells co‐transfected with Kdm7a plasmid and Sfrp1 siRNA vs the cells receiving Kdm7a plasmid and control siRNA (Figure 7E,F).

## 4 DISCUSSION

Histone lysine(K)‐specific demethylase 7A (KDM7A) has been characterized as being capable of removing di‐methylation marks of histone H3K9 and H3K27.^30^ However, the biological function of KDM7A in cell behaviour needs to be further explored. In this study, the mRNA expression levels of Kdm7a were examined in various tissues in mice. Kdm7a was highly expressed in bone and skeletal muscle. Moreover, we found that the mRNA expression of Kdm7a increased in progenitor cells during adipogenesis and osteogenesis. The data suggest that KDM7A may play a role in adipocyte and osteoblast differentiation.

To clarify the precise role of KDM7A in cell fate decision, we tested the effects of KDM7A on osteogenic and adipogenic differentiation. The data showed that the depletion of Kdm7a in undifferentiated progenitor cells inhibited the formation of adipocytes whereas stimulated the differentiation of osteoblasts. Conversely, overexpression of Kdm7a induced formation of adipocytes whereas blocked differentiation of osteoblasts. The anti‐osteogenic function seems to be contradictory to the observation that Kdm7a expression increased during osteogenic differentiation. This pattern is similar to the case of LncRNA H19, which was induced during myoblast differentiation, but exhibited an inhibitory effect on muscle differentiation.^31^ The increase in Kdm7a during osteoblast differentiation might be interpreted as a self‐balancing mechanism of cells to limit excessive osteogenesis. Of more interest, the effect of KDM7A on differentiation was largely attenuated when the catalytically dead mutation was made. These findings provide evidences that KDM7A reciprocally regulates osteoblast and adipocyte differentiation from progenitor cells and the regulation requires its action to demethylate the histones.

The effect of methylation status on transcription depends on the exact residue targeted and the degree of methylation. Tri‐methylation of H3K4, H3K36 and H3K79 is a representative marker for euchromatin, which is loosely packed, so accessible for transcription factors. In contrast, mono‐methylation of H4K20, and di‐/tri‐methylation of H3K9 or H3K27 mark heterochromatin, which is too tightly packed to be accessed by transcription factors.^24^, ^25^ Thus H3K4me2/3 and H3K79me3 usually correlate with transcriptional activation, whereas H3K9me2/3, H3K27me2/3 and H4K20me3 are preferentially associated with transcriptional repression.^16^, ^32^, ^33^ The KDM7 subfamily members can demethylate H3K9me2/1, H3K27me2/1 and H4K20me1, creating a more permissive chromatin environment for transcription of its target genes. Our data have identified that KDM7A knock‐down in progenitor cells repressed the expression levels of C/EBPα and Sfrp1. Based on the requirement of the demethylase activity of KDM7A for its regulation of progenitor cell differentiation, we have suggested that the epigenetic mechanism for KDM7A function may involve the demethylation of H3K9me2 and/or H3K27me2 marks on the promoters of C/EBPα and Sfrp1. Thus we performed ChIP assay to investigate if KDM7A could demethylate H3K9me2 or H3K27me2 at the C/EBPα and Sfrp1 promoter. We found that the presence of KDM7A at either C/EBPα or Sfrp1 promoter was down‐regulated in KDM7A‐depleted cells, indicating that KDM7A can bind to the C/EBPα and Sfrp1 promoter. Moreover, depletion of KDM7A increased the di‐methylation marks H3K9me2 and H3K27me2 at the promoter of C/EBPα and Sfrp1. These results indicate that KDM7A may play a critical role in progenitor cell fate decision by removing H3K9me2 and H3K27me2, which in turn stimulating the expression of C/EBPα and Sfrp1.

Recently, the methylation status of histones has been demonstrated to play contributing roles in osteogenic and/or adipogenic commitment of mesenchymal stem cells. Although the role of H3K27me2 in regulating adipogenic and/or osteogenic differentiation is less investigated, H3K9me2 has been reported to be a player during adipogenesis. In pre‐adipocytes, the promoter of PPARγ is initially restricted by closed chromatin modifications, eg, H3K9me2, which shifts towards an open state as the pre‐adipocyte proceeds towards differentiation.^34^, ^35^ Histone methyltransferase (HMT) G9a‐mediated H3K9me2 modification is selectively enriched on the entire PPARγ locus. H3K9me2 and G9a levels are reduced and correlate inversely with induction of PPARγ during adipogenesis. Removal of H3K9me2 after G9a deletion enhances chromatin opening and binding of the early adipogenic transcription factor C/EBPβ to PPARγ promoter to potentiate PPARγ expression.^35^ Another lab has shown that H3K9me2 at the promoter of C/EBPα contributes to the repression of C/EBPα as well and therefore inhibits adipogenesis in 3T3‐L1 cells.^36^ As a supplement, our results demonstrated that H3K9me2 at the promoter of Sfrp1 leads to the suppression of Sfrp1. These suggest that MSC fate commitment can be regulated at multiple levels by modulating the levels of H3K9me2.

While C/EBPα is a transcription factor that directly modulates adipogenesis through interacting with the essential adipogenic transcription factor PPARγ, SFRP1 is a secreted antagonist of canonical Wnt signalling which encodes a cysteine‐rich domain similar to the WNT‐binding site of Frizzled receptors. Canonical Wnt signalling is currently considered essential for skeletal development and homeostasis through regulating differentiation of bone MSCs. It has been shown that β‐catenin promotes the progression of MSCs from osteoblastic precursor cells into more mature osteoblasts while suppressing differentiation into adipogenic and chondrogenic lineages.^37^, ^38^ To be specific, the canonical Wnt pathway up‐regulates the osteogenic regulators Runx2, Dlx5 and Osterix to promote osteogenic differentiation while it inhibits the expression of the major adipogenic regulators PPARγ and C/EBPα to suppress adipocyte formation.

Consistent to the data that KDM7A stimulated the expression of Sfrp1, we further demonstrated that KDM7A is capable of inactivating canonical Wnt signalling. We further tested whether SFRP1 mediates KDM7A regulation of adipogenesis and osteogenesis. When transfected alone, Sfrp1 siRNA inhibited formation of adipocytes whereas stimulated differentiation of osteoblasts from progenitor cells. We then performed KDM7A forced expression experiments under the background of Sfrp1 silencing. The potential of KDM7A to stimulate adipocyte formation and to inhibit osteoblast differentiation was largely attenuated when Sfrp1 was silenced. This clearly demonstrated that KDM7A may promote adipogenesis and inhibit osteogenesis partially through promoting Sfrp1 expression and inactivating canonical Wnt signalling.

In summary, our work has identified novel KDM7A functions as a reciprocal regulator of osteogenesis and adipogenesis. The function is based upon its epigenetic regulation of SFRP1 and C/EBPα. Our study provides insights into the epigenetic mechanisms underlying the lineage commitment of MSCs. These findings may contribute to new therapeutic clues for metabolic disorders such as osteoporosis.

## CONFLICT OF INTEREST

The authors declare no conflict of interests.

## AUTHOR CONTRIBUTION

Xiaoyue Yang, Guannan Wang and Yi Wang, collection and assembly of data, data analysis and interpretation, final approval of manuscript; Jie Zhou and Hairui Yuan, collection and assembly of data, final approval of manuscript; Xiaoxia Li and Ying Liu, conception and design, data analysis and interpretation, final approval of manuscript; Baoli Wang, conception and design, data analysis and interpretation, manuscript writing, final approval of manuscript.
