# Supplementary material for: Histone demethylase KDM7A reciprocally regulates adipogenic and osteogenic differentiation via regulation of C/EBPα and canonical Wnt signalling
Source: J Cell Mol Med. 2019 Jan 7;23(3):2149–62. doi: 10.1111/jcmm.14126 (PMC6378189; doi:10.1111/jcmm.14126)
Supplement: Supplementary file 1 [file JCMM-23-2149-s001.doc]

#### Supplementary Table 1. Primers used for RT-PCR and ChIP assays

| Genes | Forward primer sequences: | Reverse primer sequences |
| --- | --- | --- |
| PPARγ | CTTGACAGGAAAGACAACGG | GCTTCTACGGATCGAAACTG |
| C/EBPα | CTGATTCTTGCCAAACTGAG | GAGGAAGCTAAGACCCACTAC |
| aP2 | AAATCACCGCAGACGACAGG | GGCTCATGCCCTTTCATAAAC |
| adipsin | TGATGTGTGCAGAGAGCAAC | CGTAACCACACCTTCGACTG |
| Runx2 | TCCTGTAGATCCGAGCACCA | CTGCTGCTGTTGTTGCTGTT |
| Alp | CCAGAAAGACACCTTGACTGTGG | TCTTGTCCGTGTCGCTCACCAT |
| Osterix | GGCTTTTCTGCGGCAAGAGGTT | CGCTGATGTTTGCTCAAGTGGTC |
| Osteocalcin | GCAATAAGGTAGTGAACAGACTCC | CCATAGATGCGTTTGTAGGCGG |
| Kdm7a | CCAGAGTGAGGTGTTCTTTGGAG | CAGTCCTGAGAAGTGAGCACAG |
| Sfrp1 | CAATACCACGGAAGCCTCTAAGC | GCAAACTCGCTTGCACAGAGATG |
| C/EBPβ | CAACCTGGAGACGCAGCACAAG | GCTTGAACAAGTTCCGCAGGGT |
| Klf7 | GGAAGGATGCGAGTGGCGTTTT | CGCAAGATGGTCAGACCTGGAG |
| Klf9 | CTACAGTGGCTGTGGGAAAGTC | CTCATCCGAGCGCGAGAACTTT |
| Sfrp1(ChIP) | AGTGCTTTGGCTTTCTAATC | TACTCAAGTCTCAGCTCTGC |
| Sfrp1(ChIP) Ctrl | TGTCTGTCAGTTGAGTGTAAG | AACTTACTATCATCCCAAAG |
| C/EBPα(ChIP) | CATTCTCTCTCCAAACGCTC | CCTCTAAGTCACCCACTTCC |
| C/EBPα(ChIP) Ctrl | AGTTGTCCAAGGGTGTATGTAG | AGGTGGCCTTCTTTCCAGAC |
| β-actin | AAGACCTCTATGCCAACACAG | GGAGGAGCAATGATCTTGATC |

#### Supplementary Table 2. siRNA sequences used for gene silencing

| Genes | Sense sequences | Antisense sequences |
| --- | --- | --- |
| Kdm7a-siRNA1 | GCAUCAUGCUGUGGACAUUTT | AAUGUCCACAGCAUGAUGCTT |
| Kdm7a-siRNA2 | GCAGGGACAUACUUUGUUUTT | AAACAAAGUAUGUCCCUGCTT |
| Sfrp1-siRNA | GGCUUGUGCUGUUCCUGAATT | UUCAGGAACAGCACAAGCCTT |
